# Mesenchymal Stem Cells Modulate Albumin-Induced Renal Tubular Inflammation and Fibrosis

**DOI:** 10.1371/journal.pone.0090883

**Published:** 2014-03-19

**Authors:** Hao Jia Wu, Wai Han Yiu, Rui Xi Li, Dickson W. L. Wong, Joseph C. K. Leung, Loretta Y. Y. Chan, Yuelin Zhang, Qizhou Lian, Miao Lin, Hung Fat Tse, Kar Neng Lai, Sydney C. W. Tang

**Affiliations:** 1 Nephrology Division, The University of Hong Kong, Queen Mary Hospital, Hong Kong; 2 Cardiology Division, Department of Medicine, The University of Hong Kong, Queen Mary Hospital, Hong Kong; 3 Department of Ophthalmology, The University of Hong Kong, Queen Mary Hospital, Hong Kong; The University of Hong Kong, Hong Kong

## Abstract

Bone marrow-derived mesenchymal stem cells (BM-MSCs) have recently shown promise as a therapeutic tool in various types of chronic kidney disease (CKD) models. However, the mechanism of action is incompletely understood. As renal prognosis in CKD is largely determined by the degree of renal tubular injury that correlates with residual proteinuria, we hypothesized that BM-MSCs may exert modulatory effects on renal tubular inflammation and epithelial-to-mesenchymal transition (EMT) under a protein-overloaded milieu. Using a co-culture model of human proximal tubular epithelial cells (PTECs) and BM-MSCs, we showed that concomitant stimulation of BM-MSCs by albumin excess was a prerequisite for them to attenuate albumin-induced IL-6, IL-8, TNF-α, CCL-2, CCL-5 overexpression in PTECs, which was partly mediated via deactivation of tubular NF-κB signaling. In addition, albumin induced tubular EMT, as shown by E-cadherin loss and α-SMA, FN and collagen IV overexpression, was also prevented by BM-MSC co-culture. Albumin-overloaded BM-MSCs *per se* retained their tri-lineage differentiation capacity and overexpressed hepatocyte growth factor (HGF) and TNFα-stimulating gene (TSG)-6 via P38 and NF-κB signaling. Albumin-induced tubular CCL-2, CCL-5 and TNF-α overexpression were suppressed by recombinant HGF treatment, while the upregulation of α-SMA, FN and collagen IV was attenuated by recombinant TSG-6. Neutralizing HGF and TSG-6 abolished the anti-inflammatory and anti-EMT effects of BM-MSC co-culture in albumin-induced PTECs, respectively. *In vivo*, albumin-overloaded mice treated with mouse BM-MSCs had markedly reduced BUN, tubular CCL-2 and CCL-5 expression, α-SMA and collagen IV accumulation independent of changes in proteinuria. These data suggest anti-inflammatory and anti-fibrotic roles of BM-MSCs on renal tubular cells under a protein overloaded condition, probably mediated via the paracrine action of HGF and TSG-6.

## Introduction

Bone marrow-derived mesenchymal stem cells (BM-MSCs) with multipotent differentiation capacity and immunomodulatory properties are conceptualized as a potential therapy for tissue regeneration and organ transplantation. A variety of animal studies have shown that exogenously infused BM-MSCs can ameliorate renal dysfunction in chronic kidney disease (CKD) models. Semedo *et al.* reported an amelioration of functional parameters in rodent remnant kidney models after intravenously administered BM-MSCs, probably by modulating the inflammatory response at sites of injury [1]. In collagen 4A3-deficient mice, MSCs reduced interstitial fibrosis, though failing to delay disease progression [2]. In the UUO model, BM-MSCs treatment was conducive towards the recovery of renal function and interstitial fibrosis [3]. In STZ-induced type 1 diabetes, BM-MSCs promoted repair of injured glomeruli and prevented nephropathy [4], [5]. These studies together hold promise for applying MSCs in clinical trials in patients with CKD. However, the lack of understanding on the mechanism of action of MSCs in CKD poses a great hurdle for further development.

Most previous studies on potential mechanisms focused on the regenerative capacity of MSCs in acute kidney injury (AKI). For instance, silencing of IGF-1 in infused MSCs has been shown to abolish the beneficial effect of these cells in kidney repair by decreasing PTEC proliferation and increasing apoptosis [6]. Knockdown of VEGF reduced the effectiveness of MSCs in the treatment of ischemic AKI by decreasing tubular survival [7]. Recently, microvesicles shed by BM-MSCs were shown to completely reproduce the effect of MSCs by transferring regenerative mRNA [8]. These studies might only explain the effect of MSCs in AKI models in which renal cell death is a common phenomenon. This regenerative mechanism, however, may not adequately explain the beneficial effect of MSCs in CKD because interstitial inflammation and fibrosis are the predominant cellular events leading to organ failure. A constant feature in most forms of CKD is the presence of variable amounts of proteinuria.

We previously delineated that albumin and transferrin, the key tubulotoxic components of urine proteins, induced oxidative stress [9], C3 [10], [11], CCL-2 [12], CCL-5 [13] and IL-8 [14] in PTECs via an array of tightly regulated signaling pathway [14]. We defined tubuloglomerular [12] and glomerulotubular crosstalk pathways [15], and interaction between PTECs and infiltrating monocytes/T cells via soluble factors and direct contact during co-culture that together may amplify the tubulointerstitial inflammatory cascade by overexpressing chemokine receptors in monocytes/T cells [13]. In the diabetic milieu, exposure to high glucose, glycated albumin, and AGE intermediates stimulated a proinflammatory and profibrotic phenotype in PTECs [16]–[19]. Given the pivotal position of PTECs in the progression of CKD, we hypothesize that BM-MSCs might play an active role in modulating tubular inflammation and interstitial fibrosis under an albumin-overloaded condition. This was investigated *in vitro* using co-culture systems of PTECs and BM-MSCs, and *in vivo* in a murine model of protein overload that resembles chronic proteinuric CKD.

## Materials and Methods

### Reagents and antibodies

Renal Epithelial Cell Growth Medium (REGM) was obtained from Lonza (Walkersville, MD, USA). BM-MSCs medium was purchased from Invitrogen (Carlsbad, CA, USA). The enzyme immunoassay kit detecting IL-6, IL-8, TNF-α, CCL-2 and CCL-5 were purchased from Peprotech (Rocky Hill, NH, USA) and HGF ELISA kit, anti-HGF and anti-TSG-6 neutralizing antibodies were from R&D Systems (Minneapolis, MN, USA). Anti-NF-κB antibodies were acquired from Santa Cruz Biotechnology (Santa Cruz, CA, USA). Antibodies to phospho-p42/p44 mitogen-activated protein kinase (MAPK), phospho-IκBα (Ser32), and phospho-p38 were obtained from Cell Signaling Technology (Beverly, CA, USA). Antibodies to E-cadherin were purchased form BD Biosciences (San Jose, CA, USA). Rabbit collagen IV antibodies were obtained from Abcam (Cambridge, UK). Anti-mouse and anti-rabbit secondary antibodies were from Dako (Glostrup, Denmark).

### Cell culture

Human primary PTECs were obtained from Lonza. The cells were cultured in REGM at 37°C in 5% CO_2_ and 95% air. In all experiments, there was a ‘growth arrest’ period of 24 h in serum-free medium prior to stimulation. BM-MSCs purchased from Lonza were tested for purity by flow cytometry and for their ability to differentiate into osteogenic, chondrogenic, and adipogenic lineages by the manufacturer. Cells were positive for CD105, CD166, CD29, and CD44 and negative for CD14, CD34, and CD45. The cells were cultured according to the manufacturer's instructions. Cells within passage 3 were used for all the experiments in this study.

### BM-MSCs differentiation assay

Cells were incubated in the presence or absence of 2 mg/ml HSA for 24 h, followed by osteogenic, adipogenic and chondrogenic differentiation with StemPro differentiation kits (Invitrogen, Carlsbad, CA, USA). Osteoblasts were identified by staining with Alizarin Red S (Sigma, St Louis, MO, USA) after 21 days of incubation. The presence of adipocytes was demonstrated by staining the cytoplasmic inclusion of lipid droplet with Oil-Red-O (Sigma, St Louis, MO, USA) at 14 days of induction. Toluidine Blue Stain (Sigma, St Louis, MO, USA) was used to examine the formation of chondrogenic pellets after 14 days under differentiating condition.

### Co-culture set up and experimental conditions

Two co-culture systems were used to delineate the effect of paracrine factors secreted by BM-MSCs on tubular inflammation and EMT. In system I, PTECs were seeded onto the Transwell insert (of 0.4 µm pore size) (Corning, Cambridge, MA, USA). BM-MSCs were cultured in the lower chamber of the 12-well Transwell plate (at a MSC/PTEC ratio of 1∶5) and then co-cultured with or without 2 mg/ml human serum albumin (HSA) (CSL Bioplasma, Victoria, Australia) in upper chamber or both chambers. Basolateral side of PTECs was exposed to the paracrine factors secreted from MSCs in this system. Following 6 hours' co-culture, PTECs in the upper chamber were lysed, counted, and subjected to real-time PCR to determine gene expression (at 6 h). In system II, BM-MSCs were seeded onto the transwell insert while PTECs were cultured at the bottom of the plate at the same MSC/PTEC ratio. This system allows the apical surface of PTECs to be exposed to the soluble factors secreted by BM-MSCs. To study tubular inflammatory responses, PTEC RNA extracts and culture supernatants from the lower chamber were assayed for genes and proteins (at 6 h and 24 h, respectively) expression by real-time PCR and ELISA. For tubular EMT study, PTECs were exposed to 5 mg/ml HSA and examined for gene and protein (at 3 days and 6 days, respectively) expression by real-time PCR and Western blot in the setting of co-culture system II.

### Total RNA extraction and real-time PCR

Total RNA was extracted using Trizol reagent (Invitrogen). One microgram of total RNA was reverse transcribed to cDNA with High Capacity cDNA Reverse Transcription Kits (Applied Biosystems, Foster City, USA). Gene transcription was detected by real-time PCR in an ABI Prism 7500 sequence detection system (Applied Biosystems) using specific primers designed from known sequences in the GenBank. The primer sequences were listed in Table 1. Amplified cDNAs were analyzed by the SDS software (Applied Biosystems) and target values were normalized to β-actin mRNA using relative quantification method.

**Table 1:** Primers for quantitative real-time PCR.

Gene	Primers
Human IL-8	Forward-5′- GTG CAG TTT TGC CAA GGA GT-3′
	Reverse-5′- TAA TTT CTG TGT TGG CGC AG-3′
Human CCL-2	Forward-5′- GAT CTC AGT GCA GAG GCT CG-3′
	Reverse-5′- TGC TTG TCC AGG TGG TCC AT-3′
Human CCL-5	Forward-5′- AGA GTC CTT GAA CCT GAA C-3′
	Reverse-5′-TTG TAA CTG CTG CTG TGT-3′
Human TNF-α	Forward-5′-CTG ACA TCT GGA ATC TGG A-3′
	Reverse-5′-GTC TCA AGG AAG TCT GGA A-3′
Human IL-6	Forward-5′-TGA GAG TAG TGA GGA ACA AG-3′
	Reverse-5′-CGC AGA ATG AGA TGA GTT G-3′
Human E-cadherin	Forward-5′- GAA CGC ATT GCC ACA TAC AC-3′
	Reverse-5′- ATT CGG GCT TGT TGT CAT TC-3′
Human α-SMA	Forward-5′- ACC CAC AAT GTC CCC TCT A-3′
	Reverse-5′- GAA GGA ATA GCC ACG CTC AG-3′
Human FN	Forward-5′-CCC AAC TGG CAT TGA CTT TT-3′
	Reverse-5′- CTC GAG GTC TCC CAC TGA AG-3′
Human collagen IV	Forward-5′-CCA AGG AAG AGG TGG TGT GT-3′
	Reverse-5′-GTG CTT CAC CAG GAG GTA GC-3′
Human HGF	Forward-5′-TAC GCT ACG AAG TCT GTG-3′
	Reverse-5′-TCT TGC CTG ATT CTG TAT GA-3′
Human TSG-6	Forward-5′-GGT TGC TTG GCT GAT TAT G-3′
	Reverse-5′- GCT CAT CTC CAC AGT ATC TT-3′
Human β-actin	Forward-5′-TCC ATC ATG AAG TGT GAC GT-3′
	Reverse-5′-GAG CAA TGA TCT TGA TCT TCA T-3′
Mouse CCL-2	Forward-5′-CTC TTC CTC CAC CAC CAT-3′
	Reverse-5′- CTC TCC AGC CTA CTC ATT G-3′
Mouse CCL-5	Forward-5′-TCT ACA CCA GCA GCA AGT-3′
	Reverse-5′- TAG GAC TAG AGC AAG CAA TG-3′
Mouse collagen IV	Forward-5′-GGT CCT GTC TGG AAG AGT TT-3′
	Reverse-5′-AAA TAC AAT GGG AGG GAG AA-3′
Mouse α-SMA	Forward-5′-CTC CTC AGG ACG ACA ATC GAC A-3′
	Reverse-5′-CCT TTC CAC AGG GCT TTG TTT G-3′
Mouse β-actin	Forward-5′-TCC ATC ATG AAG TGT GAC GT-3′
	Reverse-5′-GAG CAA TGA TCT TGA TCT TCA T-3′

### Antibody-based cytokine array of BM-MSCs conditioned medium

Antibody-based cytokine array (RayBiotech, Norcross, GA, USA) was performed on 50-fold concentrated supernatants from BM-MSCs incubated with or without HSA for 24 h as previously described [20]. Quantitative human cytokines were measured using Phoretix Array (Totallab, Newcastle upon Tyne, UK) according to the manufacturer's instruction. Part of the results was confirmed by real-time PCR and ELISA.

### ELISA of proinflammatory cytokine synthesis

Growth-arrested PTECs were exposed to HSA for 24 h with or without BM-MSC co-culture. Cells culture supernatants were collected and stored at −70°C until protein assay. The IL-6, IL-8, TNF-α, CCL-2, and CCL-5 protein level in culture supernatants was determined by a commercial assay kit (Peprotech).

### Western blot analysis

Total protein was harvested with lysis buffer that contained protease inhibitor cocktails (Sigma, St Louis, MO, USA). The protein concentrations were determined by Pierce BCA method (Thermo Scientific, Rockford, IL, USA). Twenty micrograms of total protein were electrophoresed through a 12% SDS–PAGE gel before transferring to a PVDF membrane. After blocking for 1 h at RT in blocking buffer (5% BSA in TBS with 0.05% Tween-20), the membrane was incubated for 16 h with primary antibody in TBS-Tween-20. The membrane was incubated with a peroxidase-labeled secondary antibody and the antigen–antibody reaction was detected with ECL plus chemiluminescence (Amersham Pharmacia Biotech, Arlington, TX, USA).

### Immunofluorescence to detect NF-κB translocation

Nuclear translocation of NF-κB was evaluated by immunofluorescence using rabbit anti-NF-κB p65 antibody (1∶50) and FITC-conjugated goat anti-rabbit IgG (BD Biosciences). PTECs grown on cover slips were fixed with 3% paraformaldehyde, blocked with 2% BSA in 0.2% Triton ×100, and then incubated with the NF-κB p65 antibody. The slides were washed in PBS, and mounted with Vectashield plus 4′,6-diamidino-2-phenylindole (DAPI) (Vector Laboratories, Burlingame, CA, USA). Cells were visualized under a Leica microscope with the appropriate filters.

### Murine BM-MSC isolation and characterization

Mouse BM-MSCs were isolated and expanded based on published methods [21], [22]. Briefly, bone marrow cells were collected by flushing femurs and tibias with complete medium constituted with DMEM and 10% MSC qualified FBS (Invitrogen). In order to remove debris, cells were passed through a 70-µm cell restrainer (BD Biosciences) and seeded onto T75 culture flasks in suspension. To maximally eliminate any unattached hematopoietic cells, the bone marrow cells were rinsed with PBS twice after incubating for 4 h. The attached cells were cultured for 14 days. The colony forming cells were passaged once for future use. The isolated BM-MSCs were characterized with MSC marker antibodies' panel (R&D systems) by immunostaining and fluorescence microscopy. The differentiation capacity of BM-MSCs was assessed by StemPro differentiation kits (Invitrogen). Osteoblasts were identified by staining with Alizarin Red S (Sigma, St Louis, USA) after 21 days of incubation. The presence of adipocytes was demonstrated by staining the cytoplasmic inclusion of lipid droplet with Oil-Red-O (Sigma) at 14 days of induction. Toluidine Blue Stain (Sigma) was used to examine the formation of chondrogenic pellets after 14 days under differentiating condition.

### Mouse model of protein overload proteinuria

This study was approved by the Committee on the Use of Live Animals in Teaching and Research of The University of Hong Kong and was performed in accordance with the National Institute of Health *Guide for Care and Use of Laboratory Animals*. Murine protein-overload model was established in C57BL6 mice at 6 wks as previously described [23], [24]. Uninephrectomy was performed under anesthesia 5 days before BSA injections began. Low endotoxin BSA A-9430 (Sigma) was given 5 days a week intraperitoneally at 10 mg/g body weight for 4 wks. Control mice received the same volume of saline. Beginning on day 7 of BSA injection, mouse BM-MSCs (1×10^6^ cells/mouse) were injected intravenously into uninephrectomized mice with or without BSA treatment at weekly intervals until sacrifice at wk 4 of BSA injection. Renal CCL-2, CCL-5, α-SMA, collagen IV mRNA and protein expression were evaluated in 4 mice randomly selected from each group. Urinary albumin was measured by ELISA quantitation kit (Bethyl Laboratories, Montgomery, AL, USA), and blood urea nitrogen (BUN), urine and serum creatinine were determined by enzymatic method (Stanbio Laboratory, Boerne, TX, USA).

### Immunohistochemistry

Immunohistochemistry was performed as previously described in paraffin-embedded tissue sections at a thickness of 4 µm [18]. The primary antibodies used in this study were as follows: anti-CCL-2 (1∶200, Abcam), anti-CCL-5 (1∶100, Santa Cruz), anti-α-SMA (1∶100, Sigma) and anti-collagen IV (1∶400, Abcam). Sections were counterstained with hematoxylin. Positive staining were quantified by Image Pro Plus Software 5.0 (Media Cybernetics, Silver Spring, USA) and presented as IOD value.

### Statistical analysis

All data were expressed as means ± standard deviation unless otherwise specified. Statistical analysis was performed using GraphPad Prism v.5 for Windows (GraphPad Software Inc., San Diego, CA, USA). Intergroup differences for continuous variables were assessed by multivariate ANOVA. *P*<0.05 was considered statistically significant.

## Results

### MSC differentiation capacity was not altered after albumin challenge

**Figure 1:**
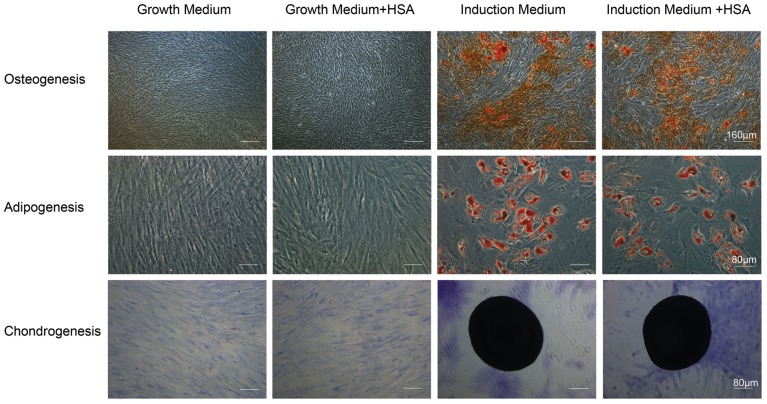
Tri-lineage differentiation capacities of BM-MSCs upon exposure to albumin. BM-MSCs were incubated with or without HSA for 24 h prior to induction of differentiation. Osteogenic differentiation was evidenced by Alizarin Red staining of mineralization (top panels) at day 21. Oil droplet stained by Oil-Red-O revealed adipogenic differentiation (middle panels) at day 14. Chondrogenic differentiation was demonstrated by toluidine blue staining of extracellular matrix formation (bottom panels) at day 14.

We first confirmed that HSA did not induce MSC differentiation. BM-MSCs were exposed to HSA (2 mg/ml). After 24-h incubation under normal growth conditions, MSCs retained their osteogenic, adipogenic and chondrogenic differentiation capacities when treated with the corresponding differentiation induction medium (Fig. 1).

### BM-MSCs attenuated albumin-induced inflammation in PTECs

**Figure 2:**
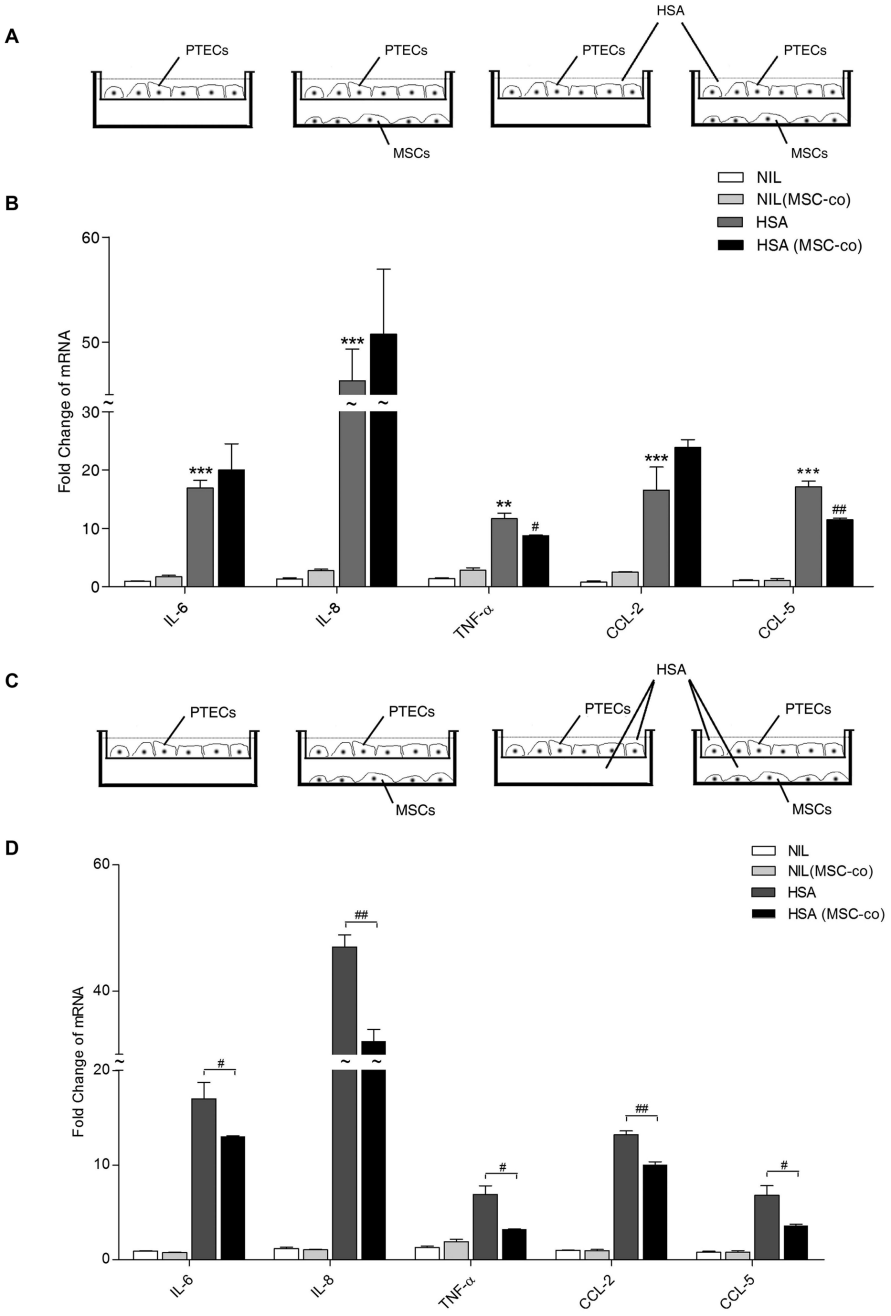
Effect of BM-MSCs on tubular inflammation in co-culture system I. (A) Co-culture set up of human PTECs with human BM-MSCs in system I. The basolateral aspect of the PTEC monolayer was exposed to MSCs. HSA (2 mg/ml) was added to the upper chamber of the Transwell insert. (B) After 6 h incubation, proinflammatory gene expression was determined by real-time PCR. Results are from three individual experiments. (C) Using co-culture system I, both chambers of the Transwell insert were supplemented with HSA. (D) Proinflammatory cytokine and chemokine expression in PTECs was assessed by real-time PCR after 6 h incubation. Results are from three individual experiments. *P<0.05, **P<0.01, ***P<0.001 versus PTECs exposed to medium control (NIL); ^#^ *P*<0.05, ^##^ *P*<0.01 versus PTECs treated with HSA.

We then tested whether BM-MSCs prevented albumin-elicited proinflammatory responses in PTECs using co-culture system I. In this system, human serum albumin (2 mg/ml) was only added to the upper chamber of the Transwell (Fig. 2A). Real-time PCR revealed that BM-MSCs significantly suppressed HSA-induced tubular overexpression of TNF-α (p<0.05) and CCL-5 (p<0.05) (Fig. 2B). When HSA was added to both chambers of the Transwell (Fig. 2C), albumin-induced IL-6, IL-8, TNF-α, CCL-2 and CCL-5 were markedly attenuated by MSC co-culture (p<0.05 for all factors, Fig. 2D).

### The immunomodulatory effect of BM-MSCs was independent of tubular polarity

**Figure 3:**
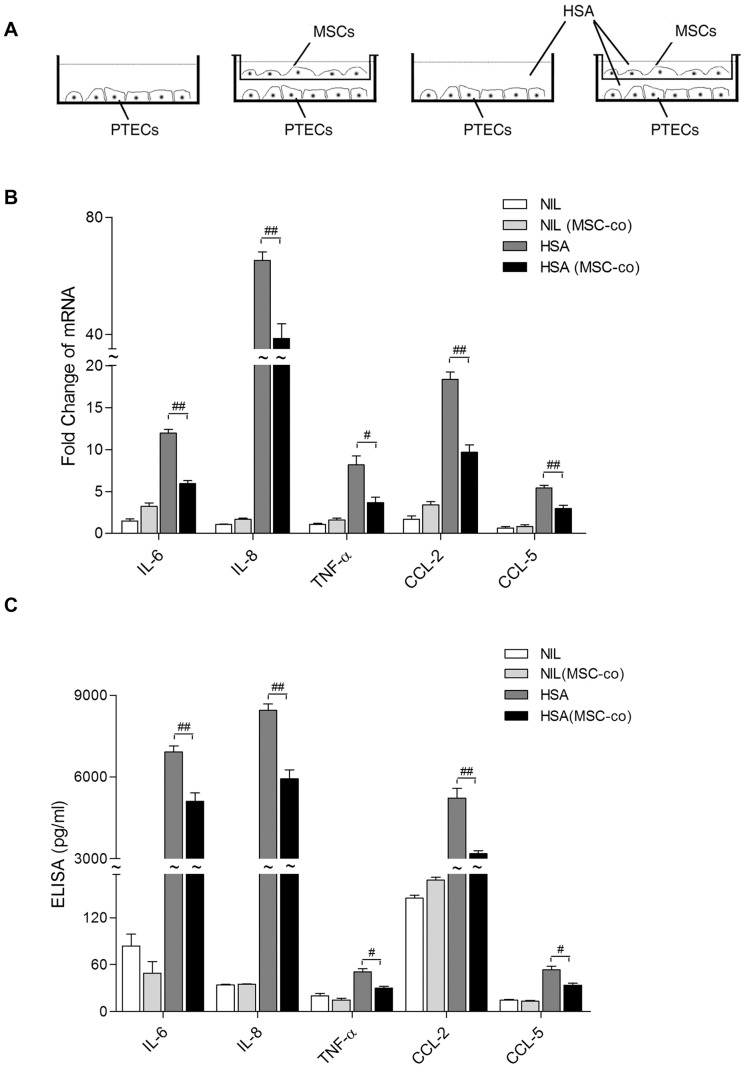
Effect of the relative orientation between BM-MSCs and PTECs. (A) Co-culture setup of human PTECs with human BM-MSCs in system II. PTECs were cultured in the lower chamber of the Transwell. MSCs were seeded (at an MSC/PTEC ratio of 1∶5) onto the Transwell insert and then co-cultured with or without the addition of HSA (2 mg/ml). The apical aspect of the PTEC monolayer was exposed to MSCs. After 6 or 24 h incubation, mRNA of the PTEC monolayer was extracted for analysis by real-time PCR (B), and supernatants from the lower chamber were collected for ELISA (C), respectively. Results are represented by means ± SD of 3–4 independent experiments. * and ** denote significant differences at *P*<0.05 and *P*<0.01, respectively.

PTECs are polarized cells that have distinct structural construction and molecular composition between apical and basolateral surfaces of the cell membrane [13], [25]. To characterize whether the polarity of PTEC might affect its response to BM-MSCs, we employed co-culture system II (Fig. 3A) in which the apical side of PTECs was exposed to BM-MSCs during co-culture. Similar to system I, HSA-upregulated proinflammatory genes in PTEC were all significantly suppressed by BM-MSC co-culture (Fig. 3B). In addition, the secretion of IL-6, CCL-2, CCL-5, IL-8 and TNF-α proteins was reduced in the presence of BM-MSCs (p<0.05) (Fig. 3C).

### The anti-inflammatory effect of BM-MSCs on PTECs was associated with attenuation of NF-κB activation

**Figure 4:**
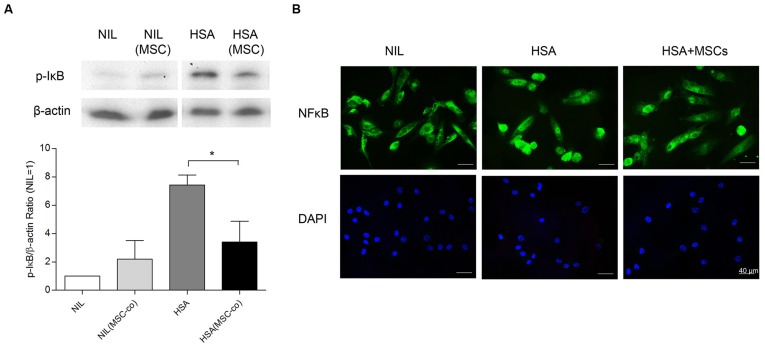
Human BM-MSCs attenuated HSA-induced phosphorylation of I-κB and nuclear translocation of NF-κB in PTECs. PTECs and MSCs were mono- and co-cultured with or without exposure to HSA (2 mg/ml) for 1 h, and the phosphorylation state of IκB in PTEC was detected by immunoblotting against anti-phospho-IκB antibody. (A) A representative Western blot. (B) Quantification. The levels of phosphorylation were normalized to actin. Results were obtained from three independent experiments. *P<0.05. (C) Representative images of NF-κB subcellular translocation. PTEC mono-culture or co-culture with BM-MSCs were incubated with HSA (2 mg/ml) for 2 h and stained by immunofluorescence for the p65 subunit of NF-κB (green, top panels) and nuclei with DAPI (blue, bottom panels).

Since NF-κB signaling was shown to mediate the tubular proinflammatory response induced by albumin overload [14], we then investigated whether BM-MSCs prevented tubular inflammation by attenuating NF-κB signaling. PTECs were incubated with HSA (2 mg/ml) for various time points in the presence or absence of BM-MSC co-culture (system II). HSA markedly upregulated the phosphorylation of I-κB in PTECs at 1 h of incubation, and this process was attenuated by BM-MSC co-culture (Fig. 4A). Furthermore, nuclear translocation of NF-κB p65 subunit induced by HSA was partially blocked by BM-MSC co-culture (Fig. 4B).

### Albumin-induced tubular EMT was suppressed by BM-MSCs

**Figure 5:**
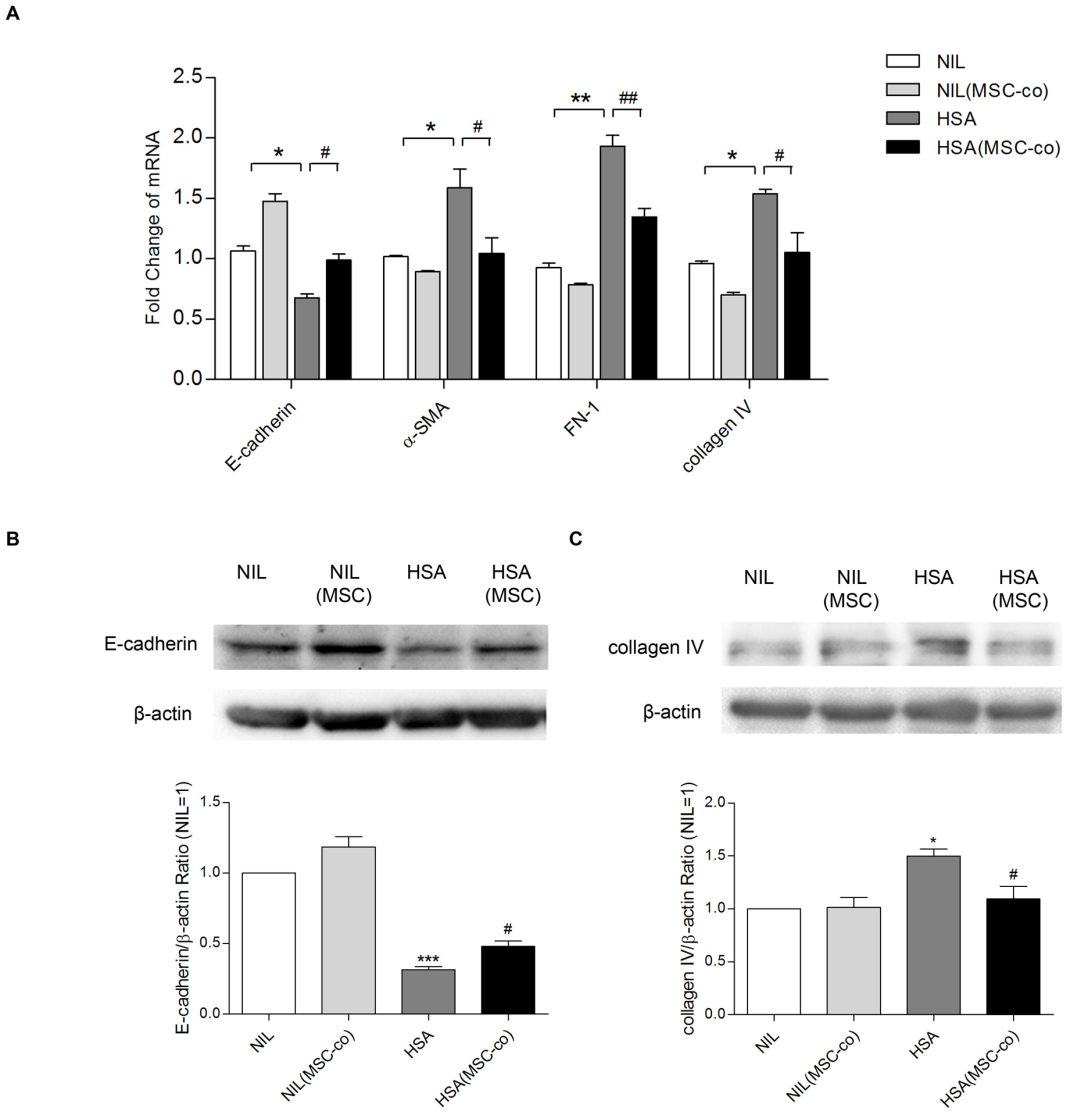
Effect of BM-MSCs on tubular EMT induced by HSA. Using co-culture system II, PTECs were co-cultured with BM-MSCs in the presence of HSA for 3 days for mRNA detection (A) and 6 days for protein examination (B and C). Results were representatives of 3 independent experiments. **P<0.05, **P<0.01, ***P<0.001 versus PTECs exposed to medium control (NIL); ^#^ *P*<0.05 versus PTECs treated with HSA.

A growing body of evidence indicated that long-term incubation with albumin was able to induce tubular EMT [26], [27]. However, the effect of BM-MSCs on albumin-induced tubular EMT has not been examined. We co-cultured PTECs with BM-MSCs for 3 days using system II in the presence or absence of HSA (5 mg/ml). BM-MSCs co-culture significantly suppressed tubular EMT as they restored E-cadherin mRNA and attenuated the upregulation of α-SMA, fibronectin (FN) and collagen IV mRNAs induced by HSA (Fig. 5A). After 6 days of co-culture, BM-MSCs also prevented the loss of E-cadherin protein and the upregulation of collagen IV protein induced by HSA (Fig. 5B and C).

### BM-MSCs were activated to release paracrine factors under albumin overload

**Figure 6:**
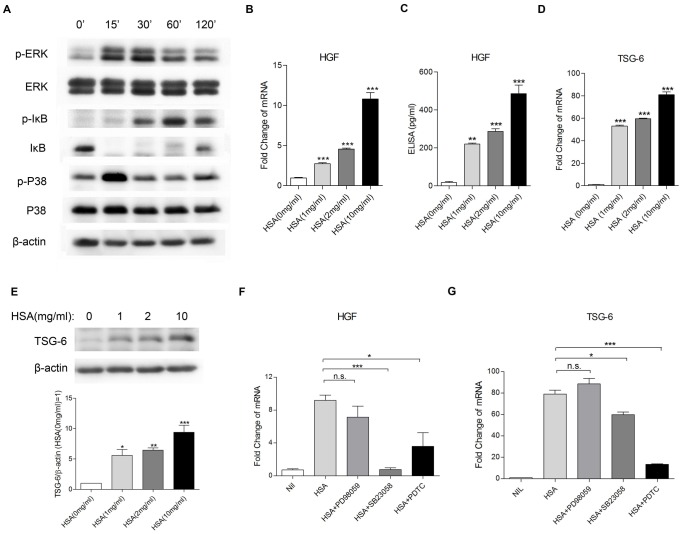
Synthesis of HGF and TSG-6 by BM-MSCs and the signaling pathways involved. (A) Western blot analysis of signaling pathways in BM-MSCs mono-culture. BM-MSCs were incubated with HSA (2 mg/ml) for 0–120 min. Total protein from BM-MSCs was collected and subjected to Western blotting for ERK, NF-κB and p38 signals. (B and C) Dose effect of HSA on mRNA and protein expression of HGF. BM-MSCs were exposed to increasing doses of ambient albumin (from 1 to 10 mg/ml) for 6 h (for real-time PCR) or 24 h (for ELISA assay). (D and E) Dose effect of albumin on mRNA and protein expression of TSG-6. BM-MSCs were incubated with HSA (2 mg/ml) for 6 h. All results represent means ±SD from 3 independent experiments. *P<0.05, **P<0.01, ***P<0.001 versus medium control. (F and G) Effect of the corresponding inhibitors on HSA-induced HGF and TSG-6 expression. BM-MSCs were treated for 6 h with medium control, HSA (2 mg/ml), the ERK inhibitor PD98059 (10 µM, added 1 h before the addition of HSA), the NF-κB inhibitor PDTC (25 µM, added 1 h before the addition of HSA) or the p38 inhibitor SB203580 (25 µM, added 1 h before the addition of HSA). Results are mean ±SD from 3 independent experiments. *n.s.* indicates no significant difference. * and *** denote significant differences at *P*<0.05 and *P*<0.001, respectively.

In search of possible paracrine factors contributing to the effect of BM-MSCs on tubular injury, an antibody-based cytokine array was utilized to screen for 507 human proteins. A total of 34 factors were apparently upregulated compared with medium control (≥2-fold increase) (Table 2). Of interest, HGF and TSG-6, both known to be important in regulating inflammation and fibrosis, were elevated under an albumin-overloaded condition. We confirmed in BM-MSC mono-culture that HSA upregulated HGF and TSG-6 expression in a dose-dependent manner (Fig. 6B–E), together with phosphorylation of p38, ERK and I-κB by Western blotting (Fig. 6A). Pre-incubation with SB203580 and PDTC but not PD98059 completely or partially inhibited the overexpression of HGF and TSG-6 in BM-MSCs exposed to HSA (Fig. 6F and G).

**Table 2:** The 34 proteins overexpressed in human BM-MSCs stimulated by HAS.

Cytokines	Normal Medium	Albumin Loaded Medium	Ratio
Activin A	397.0	896.4	2.3
CD 163	143.0	300.1	2.1
CD30/TNFRSF8	35.0	84.6	2.4
CV-2/Crossveinless-2	32.5	66.8	2.1
Endocan	77.0	201.5	2.6
Eotaxin-2/MPIF-2	25.0	51.7	2.1
FGF-11	63.5	158.9	2.5
Glut5	40.0	91.0	2.3
GRO	161.5	437.4	2.7
HGF	25.0	75.4	3.0
HGFR	142.0	289.8	2.0
IL-1 F8/FIL1 eta	48.0	101.3	2.1
IL-15 R alpha	53.0	134.1	2.5
IL-17B	61.0	149.2	2.4
IL-6	142.0	351.2	2.5
IL-8	92.0	464.4	5.0
I-TAC/CXCL11	27.5	75.4	2.7
Lipocalin-1	35.0	114.2	3.3
Lipocalin-2	25.0	54.9	2.2
Lymphotactin/XCL1	35.0	73.8	2.1
MCP-1	155.0	382.5	2.5
MFG-E8	25.0	57.6	2.3
MIP 2	32.5	327.5	10.1
MMP-1	32.5	66.8	2.1
MMP-3	25.0	72.7	2.9
MMP-8	25.0	66.8	2.7
Orexin A	25.0	57.6	2.3
Pentraxin3/TSG-14	30.0	63.6	2.1
SLPI	25.0	52.3	2.1
TCCR/WSX-1	25.0	84.6	3.4
Thymopoietin	25.0	63.6	2.5
TIMP-1	53.5	108.3	2.0
TSG-6	25.0	52.0	2.1
Ubiquitin+1	25.0	52.3	2.1

### HGF and TSG-6 contributed to the anti-inflammatory and anti-fibrotic effects of BM-MSCs

**Figure 7:**
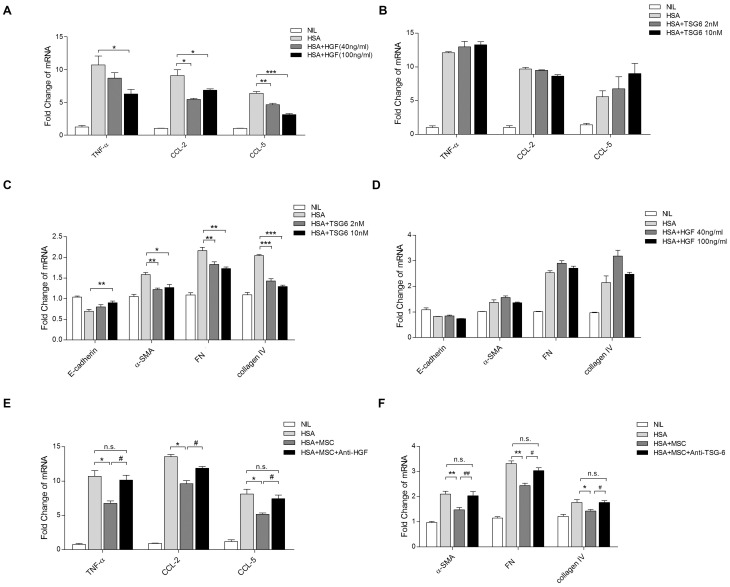
Roles of HGF and TSG-6 on HSA-induced tubular inflammation and EMT. (A–D) PTECs were pretreated with HGF and TSG-6 at different doses for 1 h. TNF-α, CCL-2, CCL-5, E-cadherin, α-SMA, FN and collagen IV mRNA expression was then determined by real-time PCR. (E and F) HGF and TSG-6 neutralizing antibodies (1 µg/ml) were introduced to PTEC and BM-MSC co-culture system II. mRNA expression of TNF-α, CCL-2, CCL-5, α-SMA, FN and collagen IV were detected by real-time PCR. Results are obtained from 3 independent experiments. *P<0.05, **P<0.01, ***P<0.001 versus PTECs treated with HSA alone; *^#^P*<0.05 versus PTECs treated with HSA and co-cultured with BM-MSCs; *n.s.*, no significant difference.

Since BM-MSC HGF and TSG-6 expression was significantly activated by HSA, we next explored the contribution of HGF and TSG-6 to the modulatory effect of BM-MSCs on tubular inflammation and EMT. Pretreatment of PTECs with recombinant HGF, but not TSG-6, for 6 h significantly attenuated HSA-induced upregulation of TNF-α, CCL-2 and CCL-5 transcripts (Fig. 7A and B). Pretreatment of PTECs with recombinant of TSG-6, but not HGF, for 3 days suppressed the upregulated expression of α-SMA, FN and collagen IV (Fig. 7C and D). Moreover, the addition of anti-HGF neutralizing antibody abrogated the suppressive effect of BM-MSCs on HSA-induced overexpression of TNF-α, CCL-2 and CCL-5 (Fig. 7E), while blocking TSG-6 by its neutralizing antibody reverted the ameliorative effect of BM-MSCs on HSA-elicited upregulation of α-SMA, FN and collagen IV in PTECs (Fig. 7F).

### Mouse BM-MSCs conferred renoprotection in murine protein overload proteinuria

**Figure 8:**
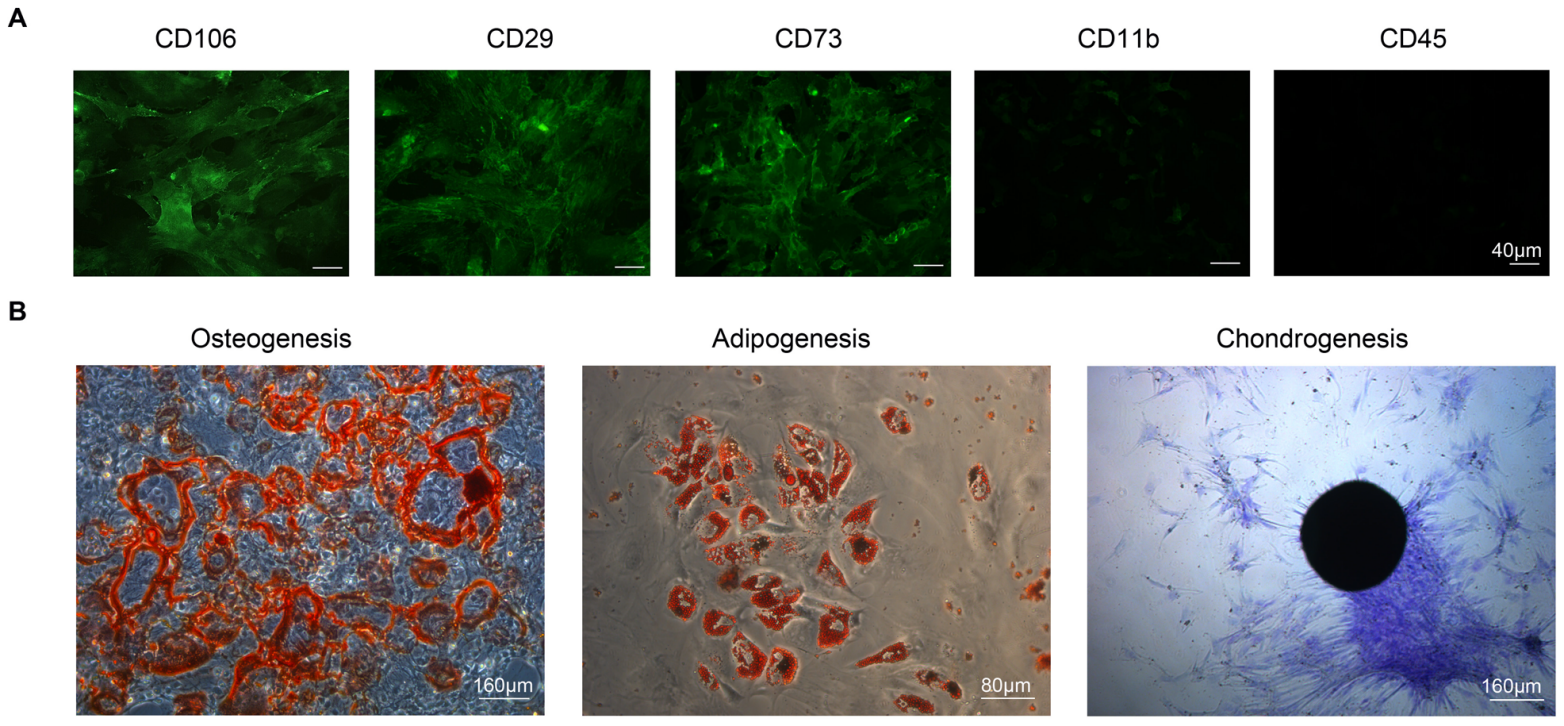
Mouse BM-MSC characterization. (A) Surface marker expression on mouse BM-MSCs. BM-MSCs were seeded on chamber slides for 24 h. Immunostaining was performed by using MSC surface marker antibodies (CD106, CD29 and CD73) and hematopoietic cell marker antibodies (CD11b and CD45). (B) Tri-lineage differentiation. Mouse BM-MSCs cultured for 14–21 days with induction medium were stained by Alizarin Red, Oil Red O and Toluidine Blue to assess osteogenic, adipogenic and chondrogenic differentiation, respectively.

To examine the effect of BM-MSCs in a murine model of protein overload proteinuria, we isolated BM-MSCs from mice femur and tibia and expanded the cells *in vitro*. These cells were characterized to be positive for CD106, CD29 and CD73, and negative for CD11b and CD45 (Fig. 8A). They also possessed tri-lineage differentiation capacity (Fig. 8B). We then created an animal model of protein overload proteinuria by daily injection of high dose of BSA (10 mg/g body weight) in uninephrectomized mice for up to 4 weeks. Repeated BSA injection for 4 wks significantly increased serum BUN and urinary albumin-to-creatinine ratio (UACR) (Table 3), indicating kidney injury in these mice. In albumin-overloaded mice that received MSC treatment, serum BUN was significantly reduced together with a trend towards a lower UACR level compared with control animals (Table 3).

**Table 3:** Physical and biochemical parameters of experimental animals (at wk 4 of BSA injection).

Parameters	UNX	UNX+MSCs	UNX+BSA	UNX+BSA+MSCs
N	7	8	9	8
Body Weight (g)	24.8±0.6	24.7±0.2	22.5±0.2^a^	23.9±0.4^b^
UACR (µg/mg)	180.6±22.4	146.1±11.7	785.2±148.9^a^	656.0.3±88.3^a^
BUN (mg/dL)	32.1±1.3	33.6±1.3	48.3±1.3^a^	29.9±1.4^b^

UNX, uninephrectomy; UACR, urine albumin-to-creatinine ratio; BUN, blood urea nitrogen.

a P<0.05 versus UNX group.

b P<0.05 versus UNX+BSA group.

### BM-MSCs attenuated tubular inflammation in protein-overloaded mice

**Figure 9:**
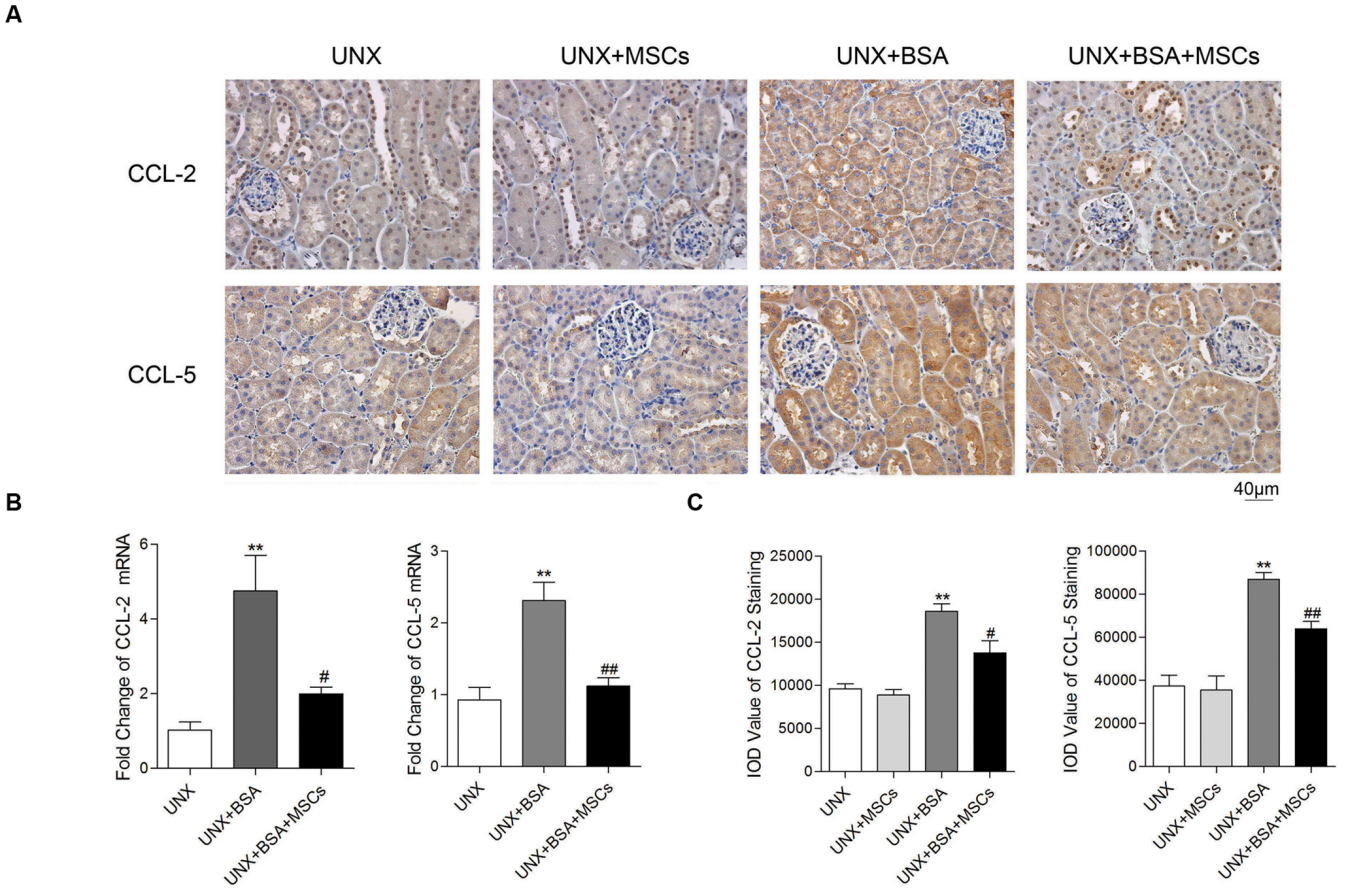
Tubular inflammation in protein-overloaded mice with or without BM-MSC treatment. (A) Cortical immunohistochemical staining for CCL-2 and CCL-5. (B) Renal cortical expression of CCL-2 and CCL-5 mRNA determined by real-time PCR. Results were from 4 mice randomly selected from each group. (C) Quantitative analysis of tubular CCL-2 and CCL-5 staining. n = 6 for UNX and UNX+MSCs, n = 7 for UNX+BSA and UNX+BSA+MSCs. **P<0.01 *versus* UNX group; *^#^P*<0.05, *^##^P*<0.01 *versus* UNX+BSA group.

To determine whether BM-MSCs influenced tubular damage in protein-overloaded mice, we examined the cortical expression of the inflammatory genes, CCL-2 and CCL-5, by real-time PCR and immunohistochemical staining. BSA treatment caused a 5-fold increase in CCL-2 mRNA expression and 2-fold increase in CCL-5 mRNA expression (Fig. 9B). These were associated with heavy tubular staining for CCL-2 and CCL-5 (Fig. 9A and C). Treatment with BM-MSCs markedly attenuated the upregulation of CCL-2 and CCL-5 mRNA expression, and the increase in CCL-2 and CCL-5 immunostaining.

### Tubulointerstitial fibrosis was suppressed by BM-MSCs treatment

**Figure 10:**
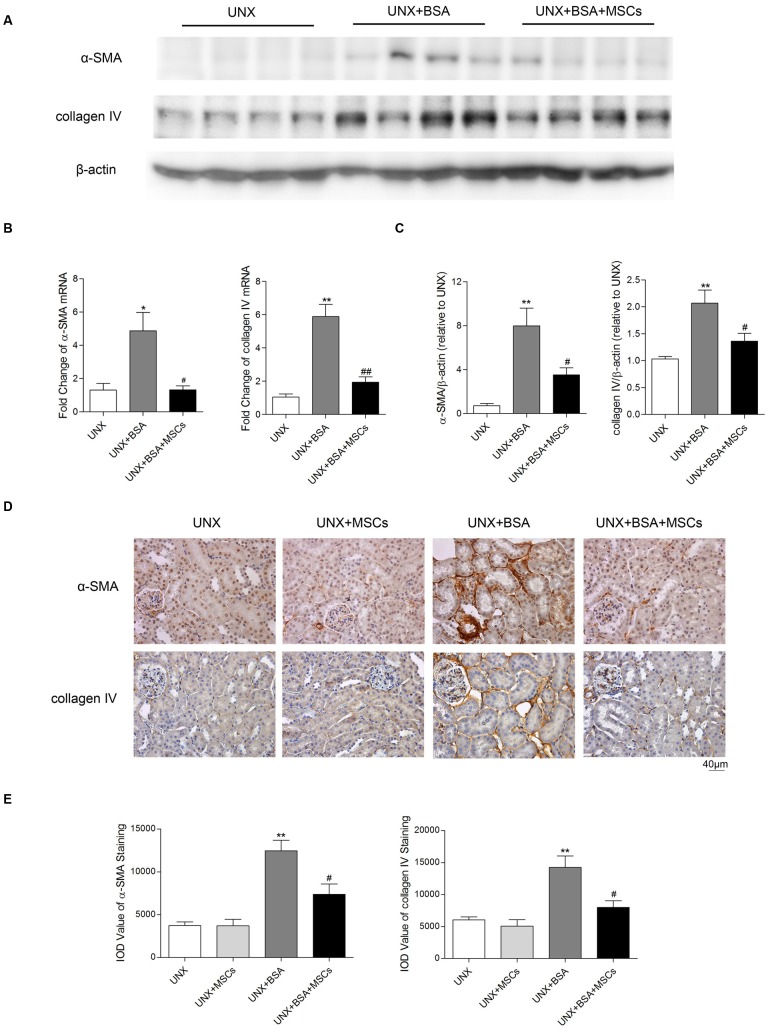
BM-MSCs reduced cortical expression of α-SMA and collagen IV. (A) Renal cortical expression of α-SMA and collagen IV mRNA determined by real-time PCR. (B) Western blot analysis of renal cortical α-SMA and collagen IV protein expression. (C) Quantitative analysis of Western blot. n = 4 mice randomly selected from each group. *P<0.05, **P<0.01 *versus* UNX group; *^#^P*<0.05, *^##^P*<0.01 *versus* UNX+BSA group. (D) Representative immunostaining of tubulointerstitial α-SMA (upper panel) and collagen IV (lower panel). (E) Quantitative analysis of α-SMA and collagen IV. n = 6 for UNX and UNX+MSCs, n = 7 for UNX+BSA and UNX+BSA+MSCs. **P<0.01 *versus* UNX group; *^#^P*<0.05 *versus* UNX+BSA group.

Interstitial fibrosis is a hallmark of the protein overload model [28]. To examine the effect of BM-MSCs on interstitial fibrosis, we detected the expression of fibrotic markers in renal cortical tissue. Protein overload induced a 4.9-fold increase in α-SMA mRNA (Fig. 10B) and an 8-fold increase in its protein level (Fig. 10A and C), which was associated with intense tubulointerstitial α-SMA immunostaining (Fig. 10D and E). BM-MSC treatment substantially prevented all these changes (Fig. 10). Similarly, collagen IV was overexpressed in the renal cortex of proteinuric mice, which was abrogated by BM-MSC treatment (Fig. 10).

## Discussion

CKD is a global health problem that afflicts millions [29]. Its prognosis is largely governed by the extent of tubulointerstitial pathology [30]. Effective protocols that could stamp or reverse its progression to ESRD are lacking. The emergence in recent years of stem cell therapy brings new hope to a variety of conditions. Its application in CKD, however, is still limited due to the complexity of the many different resident cell types within the kidney. Abnormal protein trafficking is a hallmark in many forms of CKD and impacts tubulointerstitial inflammation. Indeed, the prognostic value of proteinuria is demonstrated in numerous clinical studies among both diabetic and nondiabetic CKD [31]–[33]. Here, we sought to explore the renoprotective potential of MSCs in culture systems of proximal tubular cells and in an animal model of overload proteinuria that resembles the proteinuric state in many forms of human CKD.

We constructed a simple co-culture system that allowed the interaction between BM-MSCs and PTECs via a panel of soluble molecules under an albumin-overloaded milieu. This co-culture setup mimicked the *in vivo* environment encountered by the proximal tubule in proteinuric patients receiving BM-MSC therapy. Using this setup, we showed for the first time that BM-MSCs exerted a suppressive effect on albumin-induced proximal tubular cell inflammatory responses and epithelial-to-mesenchymal transition. As MSCs that were concomitantly stimulated with HSA produced a much more dramatic anti-inflammatory and anti-EMT response in PTECs, it can be inferred that an injury signal is essential for the induction of a tissue-repairing phenotype in MSCs. Here, we showed that the secretome of BM-MSCs acted on tubular cells and contributed to their anti-inflammatory and anti-EMT effects.

*In vivo* studies have shown that infused BM-MSCs were capable of migrating to sites of injury where they might encounter injurious signals propagated by damaged tissue [2], [5]. These signals comprised various growth factors or cytokines that could stimulate the reparatory capacity of BM-MSCs or initiate lineage differentiation of BM-MSCs. Whether such a local environment is conducive to the therapeutic effect of BM-MSCs was little known. Our data showed that the stress of albumin overload activated BM-MSCs and enhanced their anti-inflammatory and anti-fibrotic potential. HSA-stimulated BM-MSCs attenuated the activation of NF-κB signaling in PTECs when subjected to albumin overload. Importantly, albumin treatment neither induced differentiation nor impaired the tri-lineage differentiation capacity of BM-MSCs, suggesting that the beneficial effects were truly derived from the stem cells and not from differentiation of these cells into other more mature cell types.

Furthermore, HSA significantly activated several proinflammatory signaling pathways in BM-MSCs, namely P38, ERK and NF-κB. This in turn brought about upregulation of a number of paracrine factors in BM-MSCs. Among the 34 factors detected by cytokine array studies to be induced by albumin, the anti-inflammatory and anti-fibrotic factors, HGF and TSG-6, were regulated by P38 and NF-κB, as inhibition of these pathways prevented the HGF and TSG-6 responses. HGF is multipotent growth factor that exerts mitogenic, anti-inflammatory and anti-fibrotic responses on cells expressing the cognate receptor HGFR/c-Met. Functional expression of HGF contributes to BM-MSC migration, wound healing and tissue repair [34]. Overexpression of HGF in BM-MSCs enhanced their regenerative effect in acute myocardial infarction, while suppression of HGF expression reduced their efficacy [35], [36]. In the remnant kidney model, HGF treatment significantly ameliorated interstitial inflammation by modulating tubular expression of CCL-2 and CCL-5 [37], probably via disrupting NF-κB signaling [38].

Another important anti-inflammatory and anti-fibrotic factor, TSG-6, was previously shown to be upregulated in MSCs when stimulated by TNF-α *in vitro* [39] or when they were trapped in the lung as microemboli *in vivo* [40]. Activation of TSG-6 mediated the effect of BM-MSCs in myocardial infarction [40], cornea damage [41], and more recently peritoneal injury [42], via a common mechanism of inhibiting local inflammation and fibrosis.

In the present study, we showed that recombinant HGF dose-dependently attenuated HSA-induced mRNA expression of TNF-α, CCL-2 and CCL-5 in PTECs, while TSG-6 prevented tubular EMT by inhibiting upregulation of α-SMA, FN and collagen IV. In addition, these two factors at least partially contributed to the modulatory effect of BM-MSCs on tubular inflammation and EMT, as these phenomena were partially blocked by their respective neutralizing antibodies. Since BM-MSCs and PTECs were grown in a physically separated co-culture setup, it can be inferred that HGF and TSG-6 secreted by BM-MSCs acted in a paracrine manner to exert their modulatory effects on tubular inflammation and EMT. This observation might provide one mechanism for MSCs to promote renal recovery despite extremely low rates of migration to the site of injury and the absence of cell differentiation [4], [5].

On the other hand, recombinant HGF and TSG-6 failed to fully reproduce the suppressive effect of BM-MSCs on HSA-induced tubular inflammation and EMT. In our experiments, at least IL-6 and IL-8 expression was unaffected by HGF and blocking of TSG-6 failed to neutralize the protective effect of BM-MSCs on E-cadherin loss in PTECs. This implies that apart from HGF and TSG-6, other paracrine factors may be secreted by BM-MSCs which act on PTECs to dampen their inflammatory and fibrotic phenotype. The identification of these factors merits further investigation in order to fully exploit the reparatory potential of BM-MSCs.

One limitation of these *in vitro* studies is that they were confined to the interaction between BM-MSCs and PTECs. Our results may underestimate the involvement of other resident and infiltrating cells within the kidney such as immune cells that are probably also regulated by BM-MSCs and may have contributed to the observed recovery of tubular inflammation and EMT. Nevertheless, our results revealed that the effect of BM-MSCs on PTECs is at least direct and independent of cell-cell contact.

Finally, we showed in an animal model of overload proteinuria that BM-MSC treatment reduced serum BUN without significantly lowering proteinuria, indicating that the renal-repairing effect of BM-MSCs was independent of changes in proteinuria. On the other hand, downregulation of both CCL-2 and CCL-5 mRNA in the albumin-overloaded kidney suggests that albumin-induced renal inflammation was suppressed by BM-MSC treatment. Immunohistochemical staining further revealed that the suppressive effect of BM-MSCs on CCL-2 and CCL-5 protein expression mainly occurred in proximal tubular cells, which is consistent with our *in vitro* findings that BM-MSCs inhibited cytokine and chemokine expression in protein-overloaded PTECs. In addition, BM-MSCs treatment significantly attenuated the abnormally upregulated fibrotic markers such as α-SMA and collagen IV at both mRNA and protein levels in renal cortical tissue, supporting an additional antifibrotic effect of BM-MSCs. Immunohistochemical staining confirmed that tubular interstitial deposition of α-SMA and collagen IV was ameliorated by BM-MSC treatment.

In summary, we have provided novel data to support the reparatory effect of BM-MSCs in ameliorating renal tubular inflammation and fibrosis in the context of albumin overload that is seen in many forms of CKD. This is likely mediated by the paracrine action of HGF, TSG-6 and other factors secreted by BM-MSCs that together mitigated albumin-induced NF-κB activation and the downstream upregulation of inflammatory chemocytokines and EMT phenotypic changes in tubular cells, the key cell type that orchestrates tubulointerstitial inflammation and fibrosis. Our findings support the notion that apart from immune cells, PTECs might also be a direct target for BM-MSCs to exert their biologic actions, and may explain why BM-MSCs were able to ameliorate functional parameters in certain chronic diabetic and non-diabetic nephropathies. The potential anti-inflammatory and anti-fibrotic effects of BM-MSCs warrant confirmation in other animal models of CKD.
